# The Association Between Home Stay and Symptom Severity in Major Depressive Disorder: Preliminary Findings From a Multicenter Observational Study Using Geolocation Data From Smartphones

**DOI:** 10.2196/28095

**Published:** 2022-01-28

**Authors:** Petroula Laiou, Dzmitry A Kaliukhovich, Amos A Folarin, Yatharth Ranjan, Zulqarnain Rashid, Pauline Conde, Callum Stewart, Shaoxiong Sun, Yuezhou Zhang, Faith Matcham, Alina Ivan, Grace Lavelle, Sara Siddi, Femke Lamers, Brenda WJH Penninx, Josep Maria Haro, Peter Annas, Nicholas Cummins, Srinivasan Vairavan, Nikolay V Manyakov, Vaibhav A Narayan, Richard JB Dobson, Matthew Hotopf, RADAR-CNS

**Affiliations:** 1 Department of Biostatistics and Health Informatics Institute of Psychiatry, Psychology and Neuroscience King's College London London United Kingdom; 2 Data Science Analytics & Insights, Janssen Research & Development Beerse Belgium; 3 Institute of Health Informatics University College London London United Kingdom; 4 NIHR Biomedical Research Centre at South London and Maudsley NHS Foundation Trust and King’s College London London United Kingdom; 5 Health Data Research UK London University College London London United Kingdom; 6 NIHR Biomedical Research Centre at University College London Hospitals NHS Foundation Trust London United Kingdom; 7 Department of Psychological Medicine, Institute of Psychiatry, Psychology and Neuroscience King's College London London United Kingdom; 8 Teaching Research and Innovation Unit, Parc Sanitari Sant Joan de Déu Fundació Sant Joan de Déu Barcelona Spain; 9 Centro de Investigación Biomédica Red de Salud Mental Madrid Spain; 10 Faculty of Medicine and Health Sciences Universitat de Barcelona Barcelona Spain; 11 Department of Psychiatry, Amsterdam Public Health Research Institute and Amsterdam Neuroscience Amsterdam University Medical Centre, Vrije Universiteit and GGZ InGeest Amsterdam Netherlands; 12 H. Lundbeck A/S Copenhagen Denmark; 13 Janssen Research & Development, LLC Titusville, NJ United States; 14 see Acknowledgments

**Keywords:** major depressive disorder, PHQ-8, smartphone, GPS, home stay, mobile phone

## Abstract

**Background:**

Most smartphones and wearables are currently equipped with location sensing (using GPS and mobile network information), which enables continuous location tracking of their users. Several studies have reported that various mobility metrics, as well as home stay, that is, the amount of time an individual spends at home in a day, are associated with symptom severity in people with major depressive disorder (MDD). Owing to the use of small and homogeneous cohorts of participants, it is uncertain whether the findings reported in those studies generalize to a broader population of individuals with MDD symptoms.

**Objective:**

The objective of this study is to examine the relationship between the overall severity of depressive symptoms, as assessed by the 8-item Patient Health Questionnaire, and median daily home stay over the 2 weeks preceding the completion of a questionnaire in individuals with MDD.

**Methods:**

We used questionnaire and geolocation data of 164 participants with MDD collected in the observational Remote Assessment of Disease and Relapse–Major Depressive Disorder study. The participants were recruited from three study sites: King’s College London in the United Kingdom (109/164, 66.5%); Vrije Universiteit Medisch Centrum in Amsterdam, the Netherlands (17/164, 10.4%); and Centro de Investigación Biomédica en Red in Barcelona, Spain (38/164, 23.2%). We used a linear regression model and a resampling technique (n=100 draws) to investigate the relationship between home stay and the overall severity of MDD symptoms. Participant age at enrollment, gender, occupational status, and geolocation data quality metrics were included in the model as additional explanatory variables. The 95% 2-sided CIs were used to evaluate the significance of model variables.

**Results:**

Participant age and severity of MDD symptoms were found to be significantly related to home stay, with older (95% CI 0.161-0.325) and more severely affected individuals (95% CI 0.015-0.184) spending more time at home. The association between home stay and symptoms severity appeared to be stronger on weekdays (95% CI 0.023-0.178, median 0.098; home stay: 25th-75th percentiles 17.8-22.8, median 20.9 hours a day) than on weekends (95% CI −0.079 to 0.149, median 0.052; home stay: 25th-75th percentiles 19.7-23.5, median 22.3 hours a day). Furthermore, we found a significant modulation of home stay by occupational status, with employment reducing home stay (employed participants: 25th-75th percentiles 16.1-22.1, median 19.7 hours a day; unemployed participants: 25th-75th percentiles 20.4-23.5, median 22.6 hours a day).

**Conclusions:**

Our findings suggest that home stay is associated with symptom severity in MDD and demonstrate the importance of accounting for confounding factors in future studies. In addition, they illustrate that passive sensing of individuals with depression is feasible and could provide clinically relevant information to monitor the course of illness in patients with MDD.

## Introduction

The World Health Organization ranks depression as the single largest contributor to global disability [1]. People with major depressive disorder (MDD) often experience physical comorbidity [2], loss of occupational function [3], and low quality of life [4]. Furthermore, MDD is strongly associated with suicidal deaths and premature mortality [5]. The process for MDD diagnosis and evaluation of symptom severity is highly dependent on the subjective information that an individual under screening provides to a clinician, and it might be affected by recall bias.

Recent advances in digital technologies, including smartphones and wearable devices, enable the collection of a variety of data streams that can be used to objectively characterize an individual’s daily activity and physical condition [6]. These data can be collected continuously, remotely, and unobtrusively without affecting an individual’s daily routine and behavior. Importantly, analysis of such data could result in the development of new objective, quantifiable, cost-effective, and viable digital biomarkers of an individual’s behavioral, cognitive, and emotional states [7-9]. Once developed and thoroughly tested, digital biomarkers hold great promise for improving the diagnosis and prognosis of a variety of mental health disorders, for facilitating continuous monitoring of individual well-being, and for supporting initiatives in precision medicine by helping to establish digital patient phenotypes in different disease areas [10].

Several recent studies have demonstrated the association between MDD symptoms and mobility patterns derived from mobile devices. For example, individuals with greater severity of MDD symptoms were reported to make fewer transitions between locations of interest (ie, those frequently visited in the past) and spend more time at home [11-15]. Home stay, an indicator of social disengagement [12], has also been reported to be significantly related to the severity of MDD symptoms [12-14].

Most studies used small and homogeneous cohorts of participants (eg, university students) and were conducted over a short period (eg, several weeks). In this study, we examined the association between the overall severity of MDD symptoms and a measure of daily mobility patterns using data from a larger and more diverse group of participants collected in the Remote Assessment of Disease and Relapse–Major Depressive Disorder (RADAR-MDD) study [16]. The RADAR-MDD study is an observational, longitudinal, prospective study that is currently being conducted at multiple clinical sites spread across several European countries and is part of a wider research program (Remote Assessment of Disease and Relapse–Central Nervous System [17]) to explore the potential of wearable devices to help prevent and treat depression, multiple sclerosis, and epilepsy. We used the 8-item Patient Health Questionnaire (PHQ-8; [18]) total score to measure the severity of MDD symptoms and home stay to describe an individual’s daily mobility pattern. Home stay was selected as an interpretable measure of mobility with previous evidence suggesting that it is related to the severity of MDD symptoms [12-14]. In addition, we examined whether the strength of the relationship changes from weekdays to weekends (ie, modulated by changes in daily routine) and can be affected by an individual’s demographics and quality of the acquired GPS recordings. We hypothesized that higher levels of MDD, as quantified by the PHQ-8, would correspond to a prolonged home stay. In addition, we anticipated that the relationship between the severity of MDD symptoms and home stay would be modulated by changes in daily routine from weekdays to weekends [14]. If the hypotheses were proved to be true, this would provide additional evidence on the use of geolocation data in digital phenotyping [10].

## Methods

### Study Population

Participants were recruited for the RADAR-MDD study from November 2017 to November 2019. The recruited participants were aged ≥18 years and had experienced at least two episodes of MDD in their lifetime, with the most recent episode occurring within the last 2 years. The exclusion criteria included lifetime history of bipolar disorder; schizophrenia; MDD with psychotic features, schizoaffective disorders; history of moderate to severe drug or alcohol dependence within 6 months before enrollment; history of a major medical disease that could affect the participant’s ability to be involved in normal daily activities for >2 weeks; dementia; and pregnancy. No limitations were applied regarding any treatment that the participants were receiving over the course of the study. Written consent was obtained before the enrollment session, followed by collection of sociodemographic, social environment, and medical history and technology use questionnaires and the Lifetime Depression Assessment Self-Report [19]. Participants with MDD were recruited from three clinical sites: King’s College London (KCL) in the United Kingdom; Vrije Universiteit Medisch Centrum (VUMC) in Amsterdam, the Netherlands; and Centro de Investigación Biomédica en Red (CIBER) in Barcelona, Spain (Multimedia Appendix 1, Table S1 and Table 1). More details on the study protocol can be found in the study by Matcham et al [16].

**Table 1:** Data set characteristics (N=164).

Characteristic		Clinical site			
		KCL^a^	CIBER^b^	VUMC^c^	All sites
Participants with both PHQ-8^d^ and GPS data collected, n (%)		232 (57.9)	116 (28.9)	53 (13.2)	401 (100)
**Participants with biweekly segments fulfilling the selection criteria, n (%)**		109 (66.4)	38 (23.2)	17 (10.4)	164 (100)
	Female, n (%)	83 (76.1)	26 (68.4)	14 (82.4)	123 (75)
	Age (years), median (range; SD)	46 (18-73; 15.0)	54 (27-71; 9.8)	33 (19-69; 14.9)	48 (18-73; 14.7)
**Biweekly segments analyzed, n (%)**		483 (62.8)	222 (28.9)	64 (8.3)	769 (100)
	For employed participants	277 (72.5)	64 (16.8)	41 (10.7)	382 (100)
	For unemployed participants	204 (53)	158 (41)	23 (6)	385 (100)

^a^KCL: King’s College London.

^b^CIBER: Centro de Investigación Biomédica en Red.

^c^VUMC: Vrije Universiteit Medisch Centrum.

^d^PHQ-8: 8-item Patient Health Questionnaire.

The number of participants and biweekly segments collected at each site was normalized by the corresponding total obtained by pooling data across the 3 sites (column *All sites* in Table 1), with the resulting percentages indicated in parentheses. In all, 2 biweekly segments from KCL had no data on occupational status.

### Data Collection

We used the RADAR-based platform for data collection and storage [20,21]. Participants with MDD were required to install several apps on their smartphones. Participants without a smartphone or with a non-Android device were provided with an Android smartphone and were required to use it throughout the study [16]. Remote monitoring technology (RMT) apps were used to collect data on participant severity of experienced MDD symptoms, self-esteem, cognitive functioning, voice audio sampling, and brief in-the-moment assessments of daily life experiences. Specifically, every 2 weeks, the participants were requested to fill in the PHQ-8 in the RADAR-base active RMT app. The request notifications were sent out at a calendared time and remained active only on that day initially. The completion window was increased to 3 days to improve the completion rates in April 2019.

**Figure 1:**
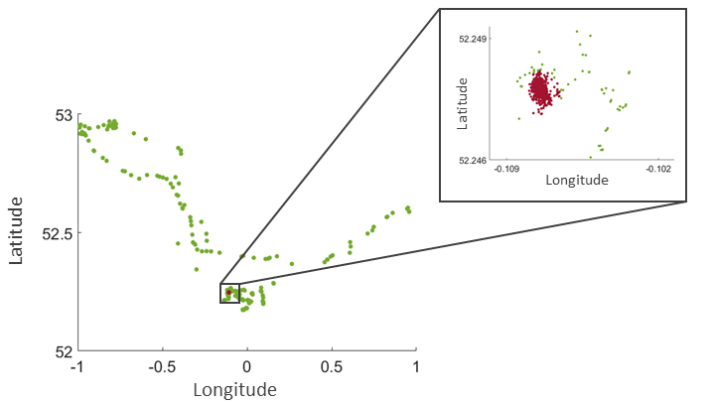
Exemplar geolocation data which correspond to a biweekly segment of a study participant. The red dots denote individual’s home location, whereas longitude and latitude along the axes are expressed in decimal degrees.

The passive RMT apps ran in the background and required minimal input from the participants. The apps collected data on participants’ physical (eg, transitions in space) and socially relevant activity (eg, number and duration of phone calls) as well as on some ambient factors (eg, ambient noise and light). GPS location data were obfuscated by adding a fixed random number to the latitude and longitude of all GPS data points generated by a single participant (Figure 1). The accuracy of each acquired GPS data point, as provided by either a mobile network operator or GPS satellites, corresponded to 1 SD (ie, radius) of a bivariate normal distribution with equal variances along the 2 spatial dimensions centered at that point. GPS data points with an accuracy >20 meters were discarded from the analyses. This accuracy level allowed the inclusion and analysis of most of the generated biweekly segments while ensuring high accuracy of the GPS recordings (Multimedia Appendix 1, Table S2). The sampling period of the GPS signal was set to either 5 minutes [13,14,22] or 10 minutes [23-25] throughout the study. However, the effective sampling period varied over time towing to occasional signal loss and battery drain (ie, signal undersampling). Other factors affecting the sampling period included occasional concurrent and asynchronous acquisition of geolocation data from both a mobile network operator and GPS satellites (ie, signal oversampling).

A single completed PHQ-8 combined with the GPS data acquired over the 2 preceding weeks and obtained from the same participant is herein referred to as a *biweekly segment*. To ensure a high quality of the analyzed geolocation data, only biweekly segments that met the following criteria were analyzed: 14 days of GPS recordings available, a daily median sampling period of the GPS signal ≤11 minutes, and the daily number of acquired GPS data points ≥48. The cutoff values were selected to maximize the volume of data available for analysis while preserving the high quality of these data (Multimedia Appendix 1, Tables S2-S4). Participants who declared their occupational status as a volunteer, student, caregiver, full- or part-time employee, or self-employed person were considered *employed*, whereas all other participants were considered *unemployed*. To avoid interference from the effect of the COVID-19 pandemic, only data collected before January 1, 2020, were analyzed.

For each day in a biweekly segment, we computed the number of GPS data points collected for each of the 24 hours separately and over the entire day. Ideally, a GPS signal sampled uniformly over a period of 5 minutes would give 12 GPS data points per hour and a total of 288 GPS data points per day. We specified completeness of the daily data as a ratio between the actual number of GPS data points collected over a day and the expected number as determined by a sampling period (ie, 288 and 144 GPS data points for the sampling periods of 5 minutes and 10 minutes, respectively). The extreme completeness values of 0.0 and 1.0 correspond to an empty and a complete day of GPS recordings, respectively, with the values in-between corresponding to partial or interrupted GPS recordings throughout a day (Multimedia Appendix 1, Table S5). Multimedia Appendix 1, Figure S1 shows the median data completeness for each hour in a day across the analyzed biweekly segments. Similarly, we divided the actual number of GPS data points collected per hour by the expected number and computed the SD of these 24 normalized values to characterize the sampling constancy of the daily data. Any positive value for sampling constancy indicates fluctuations in the volume of GPS data acquired throughout the day, with greater values indicating greater fluctuations (Multimedia Appendix 1, Table S6). Median completeness and median sampling constancy of the daily data, as computed across 14 days of a biweekly segment, were used to characterize the quality of GPS recordings acquired for that segment.

### Home Stay

Home location was identified in a stepwise manner. Initially, the home location was approximated by the median longitude and latitude of all GPS data points in a biweekly segment acquired between 12 AM and 6 AM [12-14]. To account for accidental travel and outdoor stay, all GPS data points acquired during these hours and separated by >60 meters (=3 × the accuracy level of ≤20 meters; see the *Data Collection* section) from the initial home location were discarded. The home location was finally determined as the median longitude and latitude of all remaining GPS data points (Figure 1). The distance between any 2 GPS data points *i* and *j* was computed using the Haversine WGS84 formula as follows [22]:

distance = sin^2^(Δϕ/2) + cos ϕi cos ϕj sin^2^(Δλ/2)

where *ϕ* and *λ* correspond to the latitude and longitude, respectively.

The home stay for a given day was specified as the ratio between the number of GPS data points separated by ≤60 meters from the home location and the total number of GPS data points acquired on that day. Home stay values of 0 (or 0%) and 1 (or 100%) correspond to an entire day spent outside versus at home, respectively. Median home stay, as computed across 14 days of a biweekly segment, was used to characterize the home stay of a study participant for that segment (Multimedia Appendix 1, Table S7).

### Statistical Analysis

A linear regression model was selected to test the relationship between home stay and overall severity of MDD symptoms. Specifically, home stay was used as a dependent variable, with PHQ-8 total score being used as an independent variable. Participant age at enrollment, gender (men vs women), occupational status (employed vs unemployed), median completeness, and sampling constancy of the daily data in a biweekly segment were included in the model as additional explanatory variables:

home stay≈PHQ-8 score+age+gender+occupational status+data completeness+sampling constancy

We chose home stay as a dependent variable to test its relationship not only to the severity of MDD symptoms but also to participants’ demographics and quality characteristics of the collected geolocation data in a single model in a uniform manner. For each study participant, we randomly selected one of the biweekly segments generated by that participant. The model was fitted using data from biweekly segments pooled across the participants. To obtain a CI for each of the 6 regression coefficients, the procedure of random selection of a biweekly segment per participant followed by pooling data across the participants and fitting the model was repeated 100 times. A model variable was deemed to be significantly related to home stay if a 95% 2-sided CI obtained for the regression coefficient of that parameter did not include 0. The model was fitted using data from all 3 sites combined (Table 2) and each clinical site separately (Multimedia Appendix 1, Table S8).

**Table 2:** CIs and medians for the 6 regression coefficients of the linear regression model.

Analyzed time frame	Value, median (95% CI)					
	Age	Gender	PHQ-8^a^ total score	Occupational status	Median completeness of the daily data	Median sampling constancy of the daily data
Over the entire week	*0.241 (0.161 to 0.325)* ^b^	–0.121 (–0.272 to 0.024)	*0.100 (0.015 to 0.184)*	–*0.448 (–0.631 to –0.279)*	–0.044 (–0.108 to 0.022)	–0.064 (–0.130 to 0.005)
Weekdays only	*0.254 (0.188 to 0.329)*	–0.061 (–0.220 to 0.058)	*0.098 (0.023 to 0.178)*	–*0.495 (–0.664 to –0.354)*	–0.024 (–0.097 to 0.034)	–0.041 (–0.110 to 0.041)
Weekends only	*0.148 (0.029 to 0.240)*	–0.036 (–0.265 to 0.174)	0.052 (–0.079 to 0.149)	–*0.323 (–0.535 to –0.127)*	0.023 (–0.074 to 0.101)	–0.075 (–0.144 to 0.006)

^a^PHQ-8: 8-item Patient Health Questionnaire.

^b^CIs that do not include 0 are italicized. The regression coefficients obtained with standardized data for each clinical site separately are reported in Multimedia Appendix 1, Table S8.

The model was fitted using standardized data pooled across the 3 sites for each analyzed time frame separately. The positive sign of the regression coefficients that correspond to the categorical variables (ie, gender and occupational status) indicates greater home stay for men and employed as compared with women and unemployed participants, respectively. All reported CIs are 95% 2-sided intervals.

To test whether the relationship between home stay and the independent variables differed between weekdays and weekends, a similar approach was followed. Specifically, home stay, median completeness, and sampling constancy of the daily data in a biweekly segment were estimated separately for weekdays and weekends. As a single biweekly segment included 10 weekdays and only 4 weekend days, we used 4 days to generate those estimates to equalize variance in the estimates of weekdays and weekends. For each analyzed biweekly segment, we randomly drew 4 weekdays 100 times. The medians of the estimates computed for each of these 100 draws were used to characterize the weekdays of that segment in the model.

To account for nonnormality of both dependent and independent variables as well as for differences in their variance, each variable (except for gender and occupational status) was standardized by applying the Yeo–Johnson transformation followed by the zero-mean, unit-variance normalization. All models and findings reported throughout the manuscript were obtained by using these *standardized* data. However, qualitatively similar results were obtained when using the original, nonstandardized data (Multimedia Appendix 1, Table S9). All statistical analyses were performed using the Matrix Laboratory R2019b.

## Results

### Data Set Characteristics

**Figure 2:**
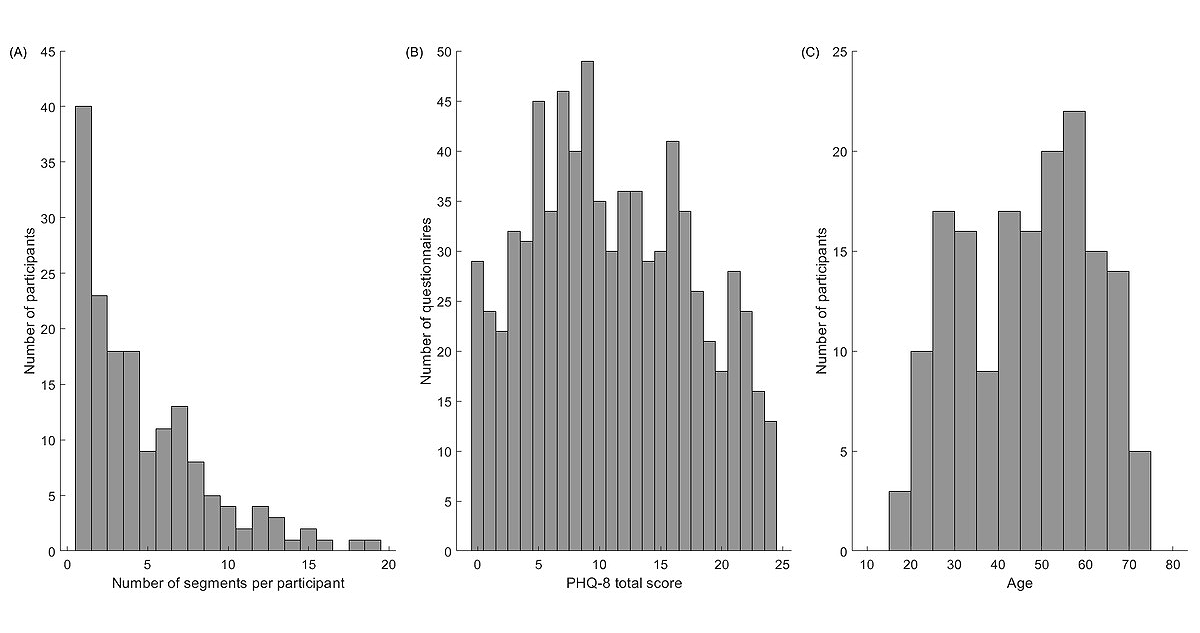
Distributions of data set characteristics. (A) Number of biweekly segments available for analysis per study participant. (B) 8-item Patient Health Questionnaire total score. (C) Participant age. Data were pooled across the 3 clinical sites. PHQ-8: 8-item Patient Health Questionnaire.

As of January 1, 2020, the total number of participants enrolled in the RADAR-MDD study across the 3 clinical sites was 432 (Multimedia Appendix 1, Table S1). Of those 432, a total of 401 (92.8%) participants had usable PHQ-8 and geolocation data (Table 1), resulting in a total of 4273 biweekly segments generated across the sites (Multimedia Appendix 1, Table S2). After discarding GPS data points with low accuracy (>20 meters) and selecting only biweekly segments with 14 days of GPS recordings available, the number of biweekly segments reduced to 43.9% (1876/4273; Multimedia Appendix 1, Table S2). Imposing additional requirements on the daily median sampling period (≤11 minutes; Multimedia Appendix 1, Table S3) and the daily minimum volume (≥48 data points; Multimedia Appendix 1, Table S4) of the GPS data in a single biweekly segment further reduced the number of biweekly segments available for analysis to 17.99% (769/4273; Table 1). The latter corresponds to data from 38% (164/432) of study participants. Table 1 lists the demographic characteristics of the participants, whereas Figure 2C shows the distribution of their ages. Most of the study participants enrolled at each clinical site were women (range 26/38, 68%-14/17, 82%; Table 1; [26]).

The number of biweekly segments available for analysis varied considerably across the sites, with VUMC (64/769, 8.3% segments; Table 1) and KCL (483/769, 62.8%) providing the least and most data, respectively. The number of biweekly segments produced by a single participant varied between 1 and 19, with a median equaling 4 (25th-75th percentiles 2-7; Figure 2A). As shown in Figure 2B, the collected data represented all 5 severity categories of MDD, as specified in the PHQ-8 questionnaire [18], including *none-minimal* (PHQ-8 total score from 0 to 4; 138/769, 18% segments), *mild* (PHQ-8 total score from 5 to 9; 214/769, 27.8% of the total), *moderate* (PHQ-8 total score from 10 to 14; 166/769, 21.6% of the total), *moderately severe* (PHQ-8 total score from 15 to 19; 152/769, 19.8% of the total), and *severe* (PHQ-8 total score from 20 to 24; 99/769, 12.8% of the total). The data set characteristics for each individual site and occupational status are shown in Multimedia Appendix 1, Figures S2 and S3.

### Estimates of Home Stay

**Figure 3:**
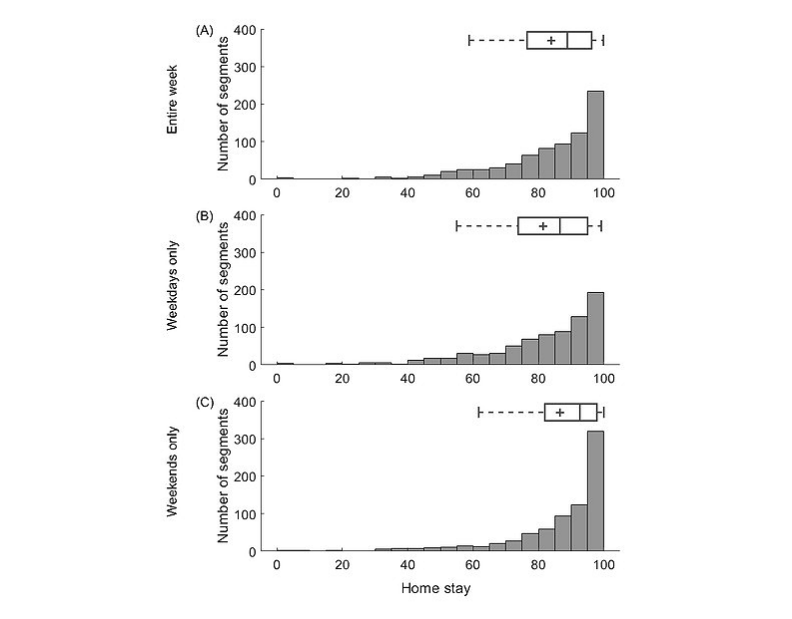
Home stay computed (A) over the entire week, (B) for weekdays, and (C) weekends only. A grey horizontal bar and a cross in each boxplot indicate median and mean of the presented data. Data were pooled across the 3 clinical sites. Home stay corresponds to the percentage of time spent at home a day.

Over the course of the study, the participants spent most of their time at home. When no distinction between weekdays and weekends was made, median home stay across the sites was 89% (21.4 hours a day; 25th-75th percentiles 76%-96% or 18.2-23.0 hours a day; Figure 3A). As expected, the home stay was lower during weekdays than during the weekends (Figures 3B and 3C). Specifically, the median home stay across the sites was 87% (20.9 hours a day; 25th-75th percentiles 74%-95% or 17.8-22.8 hours a day) and 93% (22.3 hours a day; 25th-75th percentiles 82%-98% or 19.7-23.5 hours a day) when analyzing weekday and weekend data, respectively. These observations were consistent across each clinical site (Multimedia Appendix 1, Figure S4 and Table S7).

Similarly, home stay was affected by occupational status. The employed participants spent less time at home compared with their unemployed peers. Median home stay across the sites was 82% (19.7 hours a day; 25th-75th percentiles 67%-92% or 16.1-22.1 hours a day) and 94% (22.6 hours a day; 25th-75th percentiles 85%-98% or 20.4-23.5 hours a day) for the employed and unemployed participants, respectively, with the difference being more prominent during weekdays (79% vs 93% or 19.0 vs 22.3 hours a day) than during the weekends (88% vs 96% or 21.1 vs 23.0 hours a day). The same pattern of observations was seen across each clinical site (Multimedia Appendix 1, Figure S5 and Table S7).

### Associations With Home Stay

**Figure 4:**
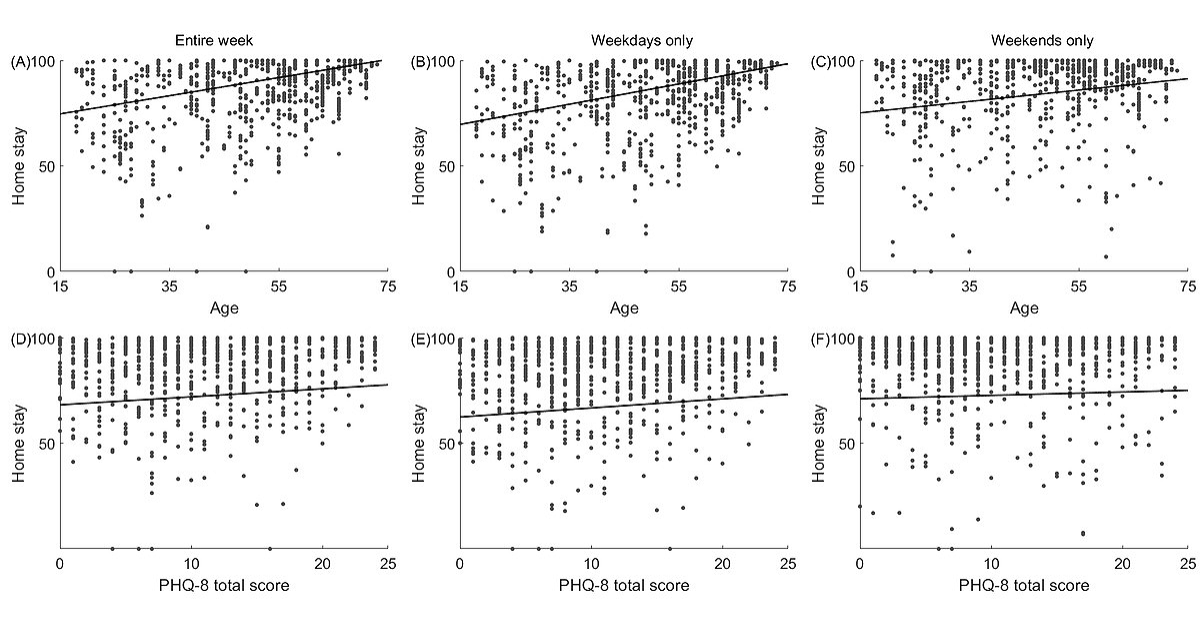
Relationship (A, B, and C) between home stay and participant age and (D, E, and F) between home stay and the 8-item Patient Health Questionnaire total score as assessed using data of (A and D) the entire week, (B and E) weekdays, and (C and F) weekends only. Each dot indicates a single biweekly segment. Data of all biweekly segments pooled across the 3 clinical sites are presented. A black line in each panel corresponds to the linear fit of the presented data. Home stay corresponds to the percentage of time spent at home a day. PHQ-8: 8-item Patient Health Questionnaire.

When data were pooled across the sites and no distinction between weekdays and weekends was made, the linear regression model revealed a significant relationship between home stay and overall severity of the MDD symptoms as captured by the PHQ-8 total score (median 0.100, 2-sided 95% CI 0.015-0.184; Figure 4D; Table 2). The latter suggested that greater overall severity of MDD symptoms was associated with prolonged home stay. The same relationship was observed when analyzing weekday data only (median 0.098, 95% CI 0.023-0.178; Figure 4E) but not on weekends (median 0.052, 95% CI −0.079 to 0.149; Figure 4F).

In addition, the model revealed a significant relationship between home stay and age. Specifically, the participants spent more time at home with age (median 0.241, 95% CI 0.161-0.325; Figure 4A; Table 2). A similar strength of the relationship was observed for weekdays (median 0.254, 95% CI 0.188-0.329; Figure 4B) and weekends (median 0.148, 95% CI 0.029-0.240; Figure 4C). Furthermore, occupational status was also found to significantly modulate home stay, with the employed participants spending less time at home compared with their unemployed peers (median −0.448, 95% CI −0.631 to −0.279; Table 2). Similar to age, there was no significant difference in the effect of occupational status on home stay among the analyzed time frames (weekdays only: median −0.495, 95% CI −0.664 to −0.354; weekends only: median −0.323, 95% CI −0.535 to −0.127).

Neither gender nor median completeness and sampling constancy of the daily data in a biweekly segment had a significant impact on home stay and this held for all the analyzed time frames (Table 2). The results of modeling for each clinical site obtained with standardized and original data are shown in Multimedia Appendix 1, Tables S8 and S9, respectively.

## Discussion

### Principal Findings

Multiple studies have demonstrated associations between patterns of daily movements of an individual in an area of the primary residence and an individual’s mood [9]. Here, we tested the association between home stay and overall severity of MDD symptoms, as reflected in the PHQ-8 total score, by using data collected in the RADAR-MDD study. The participants were invited to complete the PHQ-8 on their mobile phones every 2 weeks, whereas the same phones were used to track their geographic location continuously throughout the study. We related the PHQ-8 total score, as provided by an individual, to their median daily home stay over the 2 weeks preceding completion of the PHQ-8. In addition, we investigated how the relationship between home stay and MDD symptom severity was affected by participant age, gender, occupational status as well as by completeness and sampling constancy of the collected geolocation data. Moreover, we tested whether the strength of the relationship differed between weekdays and weekends.

The participants in the RADAR-MDD study were recruited from a nonhomogeneous population (ie, clinical and community samples with a wide age range) across 3 clinical sites in different European countries. When we pooled the data from all sites and used the entire biweekly segment before PHQ-8 completion, we found that home stay was positively associated with the PHQ-8 total score and age (Table 2). Specifically, the participants tended to spend more time at home with a greater severity of MDD symptoms and age. Furthermore, we found that occupational status was significantly related to home stay, with unemployed participants spending more time at home than their employed peers. Similar findings were observed when analyzing geolocation data collected over weekdays or weekends only, except for the association between home stay and the PHQ-8 total score (Table 2). The latter failed to reach statistical significance when tested with geolocation data of weekends only. This can be attributed to the ceiling effect [27], as the estimates of home stay obtained with geolocation data of the weekends were high for almost all participants at each individual site (Figure 3C; Multimedia Appendix 1, Figures S4 and S5 and Table S7). Although similar findings were observed for the KCL and CIBER sites, the association between home stay and the PHQ-8 total score did not reach statistical significance at the latter site (Multimedia Appendix 1, Table S8). This discrepancy could have been driven by participant recruitment primarily in a clinical setting and right skew of the PHQ-8 total scores indicating great severity of the MDD symptoms in the participants recruited at the CIBER site (Multimedia Appendix 1, Figure S2). As the VUMC site recruited only 17 participants (Table 1) that, on average, exhibited mild to moderate symptoms of MDD (Multimedia Appendix 1, Figure S2), all the findings obtained with the data of that site only should be interpreted with caution.

### Comparison With Previous Work

A variety of features can be extracted from geolocation data generated by smartphones and wearable devices and used to characterize the mobility patterns of an individual. These include home stay [15], the number of visited places [14], location entropy (ie, a metric that quantifies uniformity of the distribution of times spent by an individual at different locations) [13,14], the maximal distance from home, and the total distance traveled [22]. Remarkably, several studies that investigated the relationship between mental health disorders and mobility patterns focused on home stay features [12,22,28]. In this study, we also used home stay to quantify the mobility patterns of the study participants, as home stay is considered an important indicator of social disengagement by clinicians [12]. Moreover, it has been demonstrated that home stay has a strong negative association with location entropy [9]. No features that quantify the distance traveled between visited locations (eg, the total distance traveled or the maximal distance from home) were used in our analysis, as the notion of distance was confounded by the fact that the participants lived in both urban and rural places and in different countries.

Several previous studies have documented a positive relationship between home stay and the severity of MDD symptoms [9,13-15]. To the best of our knowledge, no study, however, has collected data from either multiple sites or a nonhomogeneous population with a confirmed clinical diagnosis of MDD. Neither did those studies thoroughly address the factors of participants’ age, occupational status, and data quality on the reported results. Furthermore, several previous studies analyzed data that were homogeneous in terms of the participant’s age (ie, student population) [12,24]. In contrast, the age of the RADAR-MDD participants ranged from 18 to 73 years (Figure 2C). Our findings demonstrate that the strength of the relationship between home stay and the severity of MDD symptoms can be modulated by age. This relationship is expected to be stronger for younger individuals and weaker for older individuals, as the latter tend to stay at home more. In addition, all RADAR-MDD participants had a clinical diagnosis of MDD, and many of them had severe symptoms of MDD, as indicated by the high PHQ-8 total scores (Figure 2B). In contrast, participants from previous studies did not undergo clinical interviews and had overall low depression scores [11-14].

It has been pointed out [9,22] that there exists no standard approach to the preprocessing of geolocation data generated by smartphones. Nonetheless, such important preprocessing steps, such as selection of an acceptable accuracy level and rate of missing data for the geolocation signal could have a significant impact on the reported results. In this study, we did not use all available geolocation data collected from the RADAR-MDD participants but instead applied stringent selection criteria (see *Data Collection*) to ensure high quality of the analyzed data and minimize the odds of reporting spurious results. In addition, we provide full and detailed information on the characteristics of the collected and analyzed geolocation data (Multimedia Appendix 1, Tables S1-S6).

### Limitations

Although this was a multicenter study and the estimates of home stay were similar across the 3 sites, most participants were recruited at KCL (Multimedia Appendix 1, Figures S4 and S5 and Table S7). Several participants in the RADAR-MDD study followed antidepressant treatment, and some of them reported comorbidity with physical illness (eg, fibromyalgia). In addition, several participants were off sick or reported ill health. Antidepressants may cause a wide range of side effects, including headaches, fatigue, weight gain, drowsiness, and dizziness [29,30]. Individuals who experience any or all of these side effects or have comorbidities with physical illness are likely to spend more time at home than outdoors. This could have inflated the reported estimates of home stay (Figure 3) and thus distorted the strength of the relationship between home stay and overall severity of MDD symptoms.

Apart from medical and mental conditions, social factors may have also influenced how much time participants spent at home. These include the number of people living under the same roof and engaging in outdoor or community activities. The participants who were expected to assist their elderly family members in daily routines or take care of their children likely spent more time at home than their peers without such responsibilities. In contrast, engagement in outdoor or community activities, such as playing bingo or going to church, likely resulted in reduced home stay. Furthermore, it is commonly assumed that employment implies the physical presence of an employee in a designated workplace outside of home. However, we cannot rule out that some employed participants worked from home. Home teleworking likely increased home stay for those participants. As employment was significantly associated with reduced home stay in our data set, most employed participants in the study still worked outside their home. The effect of medication and physical comorbidity, social factors, and home teleworking on the relationship between daily mobility patterns and severity of MDD symptoms was beyond the scope of this study, although further research is warranted.

The stringent selection criteria imposed on completeness and sampling constancy of the collected geolocation data considerably reduced the number of biweekly segments available for analysis. Several factors could have affected the quality of the collected geolocation data. Poor mobile network coverage or weak GPS signals, for example, was expected to result in a higher missing rate of geolocation recordings. This was likely the case for participants living or traveling in distant or rural areas. Smartphone battery capacity could have constrained the total duration of the geolocation recordings. Owing to a limited battery capacity, frequent user interaction with a smartphone could have accelerated the battery drain and further limited the total duration of geolocation recordings. In addition, a high number of apps running in the background could have also contributed to a more rapid battery drain. The RADAR-MDD study was designed to concurrently collect a variety of data streams (eg, from a GPS sensor, a gyroscope, an accelerometer, a microphone, and an ambient light sensor embedded in a smartphone) to characterize the individual’s behavior at full capacity [20,21]. This resulted in greater energy consumption and thus faster battery drain than in regular smartphones with no installed RADAR-MDD apps. Disabling the collection of one or multiple data streams in the study could have considerably prolonged the time smartphones operated on a single battery charge. Identification and comprehensive characterization of a single data stream or multiple data streams that convey most information on the individual’s mental well-being is still a topic of active scientific research. Alternatively, event-driven collection of all or some data streams (eg, initiated by the individual’s accidental movements or continuous motion) could have been less energy demanding than continuous sampling of those data streams as was implemented in the RADAR-MDD study. Finally, it is uncertain and requires additional examination whether the same results could have been obtained with more liberal selection criteria (eg, at least 10 instead of 14 days of recordings in a biweekly segment). If so, this would have increased the number of biweekly segments available for analysis and provided stronger evidence in support of the feasibility of geolocation data collection with smartphones.

### Conclusions

We demonstrated that longer home stay can reflect greater symptom severity in individuals diagnosed with MDD. Although the relationship between home stay and MDD severity is modest, it can nonetheless improve remote monitoring of the individual’s mental well-being, especially when combined with other informative correlates of MDD severity. However, it remains unclear whether the findings represent behavioral manifestations of MDD or are associated with changes in depressive symptoms. Additional analyses are required to test whether changes in home stay over time can be predictive of relapses in MDD. We also demonstrated that the relationship between home stay and MDD severity can be modulated by age, occupational status, and changes in daily routine. This finding is of great importance for a proper interpretation of similar studies conducted in the past and for better planning of future studies. Furthermore, our findings illustrate that passive remote monitoring of mobility patterns in individuals with MDD is feasible. This demonstrates the utility of smartphones and wearable devices with a GPS sensor in the collection of clinically relevant information that can be used to monitor the course of the disorder in a remote, unobtrusive, and ubiquitous manner, thus reducing patient burden and improving treatment.

## Appendix

Multimedia Appendix 1 Tables and figures with information for each individual population site.

## Abbreviations

CIBER: Centro de Investigación Biomédica en Red
KCL: King’s College London
MDD: major depressive disorder
PHQ-8: 8-item Patient Health Questionnaire
RADAR-MDD: Remote Assessment of Disease and Relapse–Major Depressive Disorder
RMT: remote monitoring technology
VUMC: Vrije Universiteit Medisch Centrum
